# Challenges and possibilities of public and patient involvement for early-career researchers: pedagogy, practice and data governance

**DOI:** 10.1186/s40900-026-00979-1

**Published:** 2026-09-29

**Authors:** Adam Daly, Arthur Templeman-Lilley, Isobel Walsh, Alex Neuroth, Kate Molloy, Veronica Lambert

**Affiliations:** 1 School of Psychology, Dublin City University, Dublin, Ireland; 2 DCU Centre for Possibility Studies, Dublin City University, Dublin, Ireland; 3 Independent Public Representative, Cardiff, UK; 4 DCU PPI Ignite Network, Dublin City University, Dublin, Ireland; 5 DCU Anti-Bullying Centre, Dublin City University, Dublin, Ireland; 6 School of STEM Education, Innovation and Global Studies, Dublin City University, Dublin, Ireland; 7 School of Nursing, Psychotherapy and Community Health, Dublin City University, Dublin, Ireland

**Keywords:** Public and patient involvement, Early career researcher, Pedagogy, Creative involvement strategies, Data governance

## Abstract

**Background:**

Early-career researchers face unique challenges in implementing effective and meaningful public and patient involvement (PPI) in their research. These barriers prevent early-career researchers from accessing the full benefits of effective PPI and often leads to tokenistic inclusion, which disempowers public and patient contributors. Therefore, there is a critical need to explore the possibilities of how to better support early-career researchers in achieving effective involvement of the public and/or patients.

**Main body:**

Pedagogy, practice, and data governance represent three areas of PPI that present both challenges and possible opportunities to support early-career researchers. Regarding pedagogy, PPI requires deeper integration into research education as a tool which can be utilised to enhance research across disciplines. Moreover, curriculum development and delivery must align with the tenets of the field by actively involving public and patient contributors as co-educators. Regarding practice, early-career researchers often struggle with limited resources and the difficulty of reaching diverse populations who may not benefit from traditional involvement techniques. Creative involvement strategies offer an opportunity to expand early-career researchers’ repertoire of techniques to better empower contributors. Finally, data governance is an increasingly complex yet critical consideration in PPI. Early career researchers can feel ill-equipped to navigate these complexities alongside PPI contributors. We suggest a new framework to support early-career researchers in navigating this process with PPI contributors to ensure equitable and safe data governance.

**Conclusion:**

Public and patient involvement poses many challenges to early-career researchers. However, addressing these difficulties within pedagogy, practice and data governance provides possibilities for better supporting researchers in collaborating meaningfully with PPI contributors to produce more rigorous and impactful research.

## Abstract (plain language summary)

Researchers in the early stage of their careers face many difficulties when trying to involve public and patient contributors in their research. These challenges often include a lack of formal training about how to involve patients and the public, a shortage of different ways to collaborate with diverse groups of people, and uncertainty about how to handle information ownership with public and patient contributors. These challenges make the meaningful involvement of patients and the public difficult for early-career researchers, preventing them from truly benefiting from insights that these experts by experience can offer. Having identified these challenges in our own experiences as a team of early-career researchers and a member of the public, who has acted as a public and patient involvement contributor in research for several years, we believe these challenges also present opportunities for the future of public and patient involvement. First, teaching can grow to more effectively include public and patient involvement, both as a topic to study and as a way to enhance teaching. Second, we can adopt more creative involvement strategies, ensuring patients and the public can contribute in a way that feels empowering to them. Finally, the way information is managed should be discussed openly with public and patient contributors from the very start to ensure they have a sense of ownership over the project and their intellectual contributions. By taking these steps, we hope to highlight the many possibilities for making research more inclusive and meaningful for both early-career researchers and public/patient contributors alike.

## Introduction

Research is, and fundamentally should be, for the advancement of knowledge to benefit society. However, prevailing issues within research remain: wasted resources, irrelevant priorities, and a lack of implementation [1–3], failing those who need support and squandering resources that could have been used elsewhere. For these reasons, public and patient involvement (PPI) in research is essential [4].

In this commentary, PPI is framed not as a data collection tool, but as a partnership [5]. We define this as the active collaboration between researchers and those with expertise by experience, from research ideation to dissemination [6]. Through this shared decision-making, effective PPI implementation can improve research quality and impact [7, 8]. Moreover, it positions individuals affected by the research as integral partners in its process. However, implementing PPI is not without its challenges across all academic career stages, particularly for early-career researchers.

More than ever, early career researchers face additional challenges to building careers in academia and producing meaningful PPI-inclusive research. Specifically, they must navigate an increasingly competitive job market [9], mounting pressure to demonstrate impact [10], and barriers to adopting open-science research practices [11], all alongside stress and long working hours [12, 13] during a particularly precarious phase of professional development. Moreover, the increased utilisation and reporting of PPI [14, 15], growing demands from funders [16], and an ever-expanding literature base [17, 18] demonstrate not only the enhanced expectation of PPI within research, but also the difficulty involved in meeting this additional demand. These factors compound one another, making PPI both increasingly necessary and challenging for early career researchers to integrate meaningfully in their research. Consequently, it is easy, particularly amongst early-career researchers facing these challenges, for PPI to become tokenistic, ultimately compromising research quality and disempowering contributors. This highlights the need to consider how to better support this cohort who will, in the future, set the standard for PPI conduct in research.

We suggest, based on our own experiences, that a tripartite approach may help to better scaffold PPI for early-career researchers, highlighting three areas from which PPI can be conceptualised, challenges identified, and possibilities to address these difficulties considered; Pedagogy (How we learn about collaborating with the public and patients), Practice (How we conduct collaboration with the public and patients), and Data Governance (How we manage the ownership of contributor insights and the stewardship of participant data). Although not exhaustive, we identify these three areas as vital to understanding and improving PPI for early-career researchers, to be considered by both early-career individuals themselves as well as the institutions and supervisors who guide them. In this commentary, we draw on our own experiences, existing literature, and insights from a PPI contributor to outline key challenges across these three areas, while proposing models and future possibilities to address them.

## Pedagogy and learning PPI as an early career researcher

Embedding PPI within postgraduate curricula creates significant pedagogical possibilities, shifting research training priorities from a “tokenistic and tick-box” exercise to a collaborative, reflexive, and socially accountable learning experience [19]. However, a key challenge is ensuring that training moves beyond passive learning, where PPI is viewed as a ‘nice-to-have’ extra rather than an essential requirement. To address this challenge, higher education institutions, such as University College Cork and Royal College of Surgeons in Ireland, have introduced a module co-designed by partners in Ireland’s PPI Ignite Network, signalling that PPI is not an optional add-on but a core competency for postgraduate students and early career researchers [20].

While initiatives like Ireland’s PPI Ignite Network provide examples of localised curricular implementation, they reflect a broader global landscape. For example, this aligns with international policy frameworks such as the Canadian Institutes of Health Research (CIHR) Strategy for Patient-Oriented Research [21] and the US Patient-Centred Outcomes Research Institute (PCORI) Foundational Expectations for Partnerships in Research [22], which treat patient and/or public involvement as an essential research capability and advocate for shifting the public from passive subjects to active knowledge co-creators.

To translate these international mandates into practice, it remains imperative that real-world training approaches, such as inviting PPI contributors into graduate education through seminars, analysing case studies, and facilitating peer-support groups, are embedded throughout the researcher experience. This provides early-career researchers in graduate education with immersive opportunities to work “with” rather than “on” the public. Teaching and learning activities within formal curricula should enable early-career researchers to actively participate in the construction of knowledge [23]. By moving beyond PPI as a bureaucratic exercise, a creative pedagogical approach can encourage early-career researchers to view the public not merely as subjects, but as essential partners in the knowledge-production process [24].

To achieve this, PPI curricula for early-career researchers in postgraduate courses can be enhanced through critical pedagogy and open educational practices. Together, these approaches provide the analytical lens needed to dismantle traditional hierarchies and interrogate the power dynamics and socio-political complexities that often silence marginalised voices [24, 25]. Cronin (2017) defines open educational practices as “collaborative practices that include the creation, use, and reuse of open educational resources, as well as pedagogical practices employing participatory technologies and social networks for interaction, peer-learning, knowledge creation, and empowerment of learners” [26]. By integrating these practices into teaching and learning activities curriculum developers can move beyond tokenistic involvement toward authentic co-construction, where public contributors are recognised as sources of lived expertise rather than passive data points [24, 27].

**Fig. 1:**
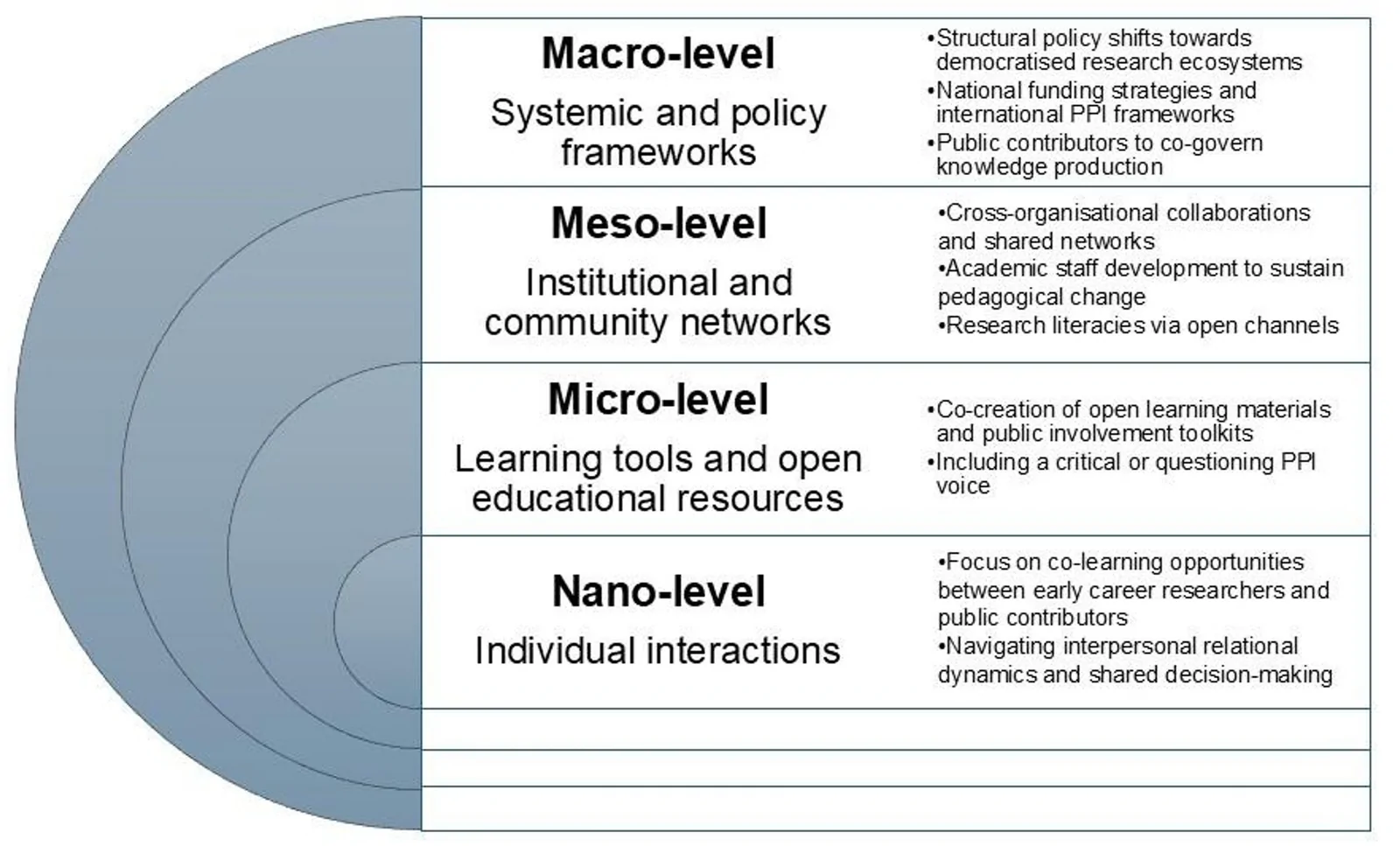
A four-level framework for embedding Patient and Public Involvement (PPI) in graduate curricula (adapted from Cronin, 2017)

To translate these pedagogical principles into practice, unpacking Cronin’s (2017) four-level framework provides curriculum developers with a structured roadmap to embed agency, structure, and culture within open educational resources and networks [26]. While PPI initiatives often focus on isolated resources, adopting Cronin’s multi-tiered approach ensures that open practice spans from individual and interpersonal relationships to systemic policy and governance (See Fig. 1).

- Nano level (individual interactions): focuses on navigating interpersonal relational dynamics and shared decision-making between early career researchers and public contributors during co-learning activities.
- Micro level (learning tools and open educational resources): centres on the co-creation of open learning materials and involvement toolkits that provide early career researchers with practical skills for public involvement. For example, a guide to working effectively with public contributors, developed by PPI experienced contributors through the National Institute for Health and Care Research (NIHR) Applied Research Collaboration (ARC) West (U.K.), emphasises the importance of “including a sometimes critical or questioning voice” [28].
- Meso level (institutional and community networks): fosters cross-organisational collaboration, open peer learning, academic staff development, and shared networks to sustain pedagogical change across postgraduate student experiences and beyond formal curricula. As a regional case example, course content co-designed by partners within Ireland’s PPI Ignite Network is available as an open digital badge via Queen’s University Belfast, with curriculum recommendations published openly on Figshare [29]. Internationally, networks like the International Patient and Public Involvement Network (IPPIN) facilitate cross-border exchange to build capacity [30]. Building on these models, Free Open Access Medical Education (FOAMed) is an example of the innovative possibilities for democratising research knowledge [31]. By leveraging open-network channels, such as social media, blogs, and podcasts, early-career researchers and public contributors can bypass traditional paywalls to access digestible learning materials [32]. This helps build the foundational research literacies needed for meaningful real-world co-creation in research design and dissemination [33]. However, while FOAMed offers a promising model for other fields to adapt it is primarily oriented towards medical and health professionals.
- Macro level (systemic and policy frameworks): open educational practices align with national funding strategies and international policy frameworks, such as the CIHR and PCORI PPI frameworks highlighted earlier, to drive structural policy shifts toward democratised research ecosystems, promoting open access infrastructure and enabling public contributors to co-govern knowledge production.

When early-career researchers, supervisors, and public contributors utilise open educational practices across these four levels, they begin to democratise PPI, ensuring that pedagogical innovations are not locked behind institutional barriers but are accessible to the communities they serve.

## Practicing PPI as an early-career researcher

High-quality PPI is a resource-intensive undertaking [34] that often feels out of reach for early-career researchers who can lack funding, networks, and experience. While traditional and economical methods of involvement in PPI, such as one-on-one or group consultations, can mitigate these challenges, an over-reliance on them can limit how lived experience is expressed and may exclude populations often labelled as underserved or less-heard [35–37]. Lacking experience with creative approaches, early-career researchers are often ill-equipped to navigate the complexities of involving diverse groups who may have additional needs as collaborators, such as Indigenous communities, children, or adults with intellectual disabilities. This is specifically in cases where these needs may not be met by traditional PPI conventions, necessitating the consideration of alternative involvement strategies. We propose that creative methodologies offer possibilities for dynamic, developmental, accessible, and cost-effective alternatives for the PPI practices of early-career researchers that could enhance both research impact and professional growth.

**Fig. 2:**
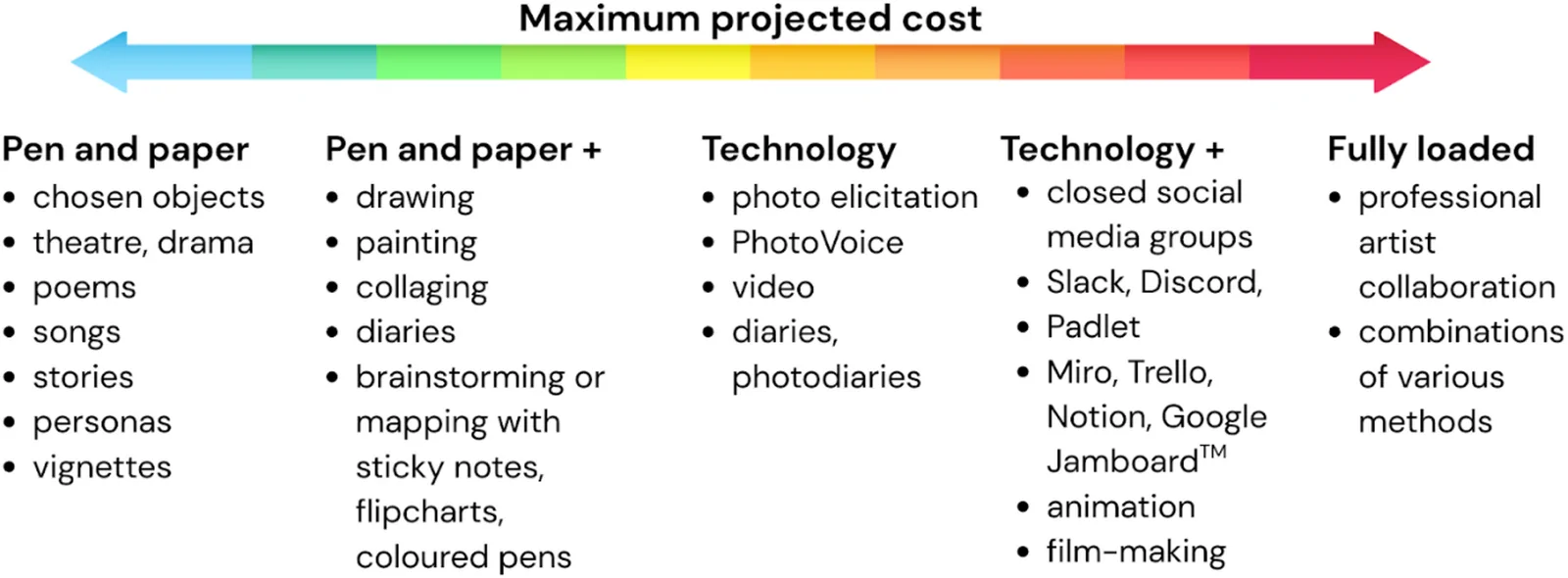
Spectrum of creative involvement strategies and relative projected costs. We acknowledge that our estimation of maximum projected costs is influenced by our own experience as researchers within a WEIRD/high-resource institutional context. Moreover, these projected costs are related only to involvement strategies that can be utilised with PPI contributor(s), and do not account for any additional remuneration costs for the contributors’ time. Involvement strategies in the ‘Pen and paper’ category can be accomplished with no more than pens, pencils and paper; strategies in the ‘Pen and paper +’ category might require extra stationary or craft materials; in the ‘Technology’ category strategies might be undertaken with feature (non-smart) phones or disposable cameras; methods in the ‘Technology +’ strategies might require the use of smartphones, laptops, or other more complex technologies; and strategies in the ‘Fully loaded’ category might require connections with arts/industry and/or sufficient funding to accommodate. For further details on individual strategies, see [35–37, 39]

Creative data collection methods, defined as novel, useful and replicable techniques used to observe or understand a given phenomenon [38], can be adopted in the PPI paradigm. In this context, these serve not as methods for data collection, but rather as methods to elicit the perspectives and involvement of contributors throughout a research project in dynamic ways, to improve accessibility by shifting power dynamics and bypassing verbal barriers [35–37]. These approaches, which we call creative involvement strategies, range in resource intensity from simple paper-based crafts to digital workspaces and collaborations with visual artists [37, 39] (See Fig. 2). For instance, participatory visual methods can allow contributors to elucidate thoughts that may be difficult to express verbally, while also granting agency to underserved populations where communication barriers exist, such as children or adults with intellectual disabilities, while mitigating the power imbalances present in traditional involvement strategies [35–37]. This approach is well-evidenced in the community-engaged, patient-oriented research process undertaken by Young and colleagues when developing the Aboriginal Children’s Health and Well-being Measure. In the development of this scale, photovoice was harnessed alongside consultations and focus groups to involve children directly in concept generation for the resulting scale [40, 41], suggesting the potential efficacy of photovoice to elucidate the thoughts of contributors on a PPI panel. Ultimately, broadening the repertoire of involvement strategies available to an early-career researcher allows for a wider diversity of expression, facilitating meaningful representation for silent or marginalised groups [35].

Furthermore, the outputs of creative involvement strategies with PPI contributors can be leveraged to educate the general public and mobilise policymakers [35]. This is exemplified by The Chimera Art and Science Programme, where scientists and artists collaborated with the CÚRAM laboratory to produce a research exhibition. The project not only resonated with the public, but also sparked creative discussions regarding future research directions [42]. A further impactful example is found in Tyler et al. (2021), where, alongside group discussions and research priority-setting activities, PPI contributors collaborated one-on-one with a patient experience artist to develop portrait artwork expressing their health concerns [43]. Beyond the research insights gained, Tyler et al. (2021) acknowledged that the main advantage of this creative method was the contributors’ belief that their experiences would be more powerful catalysts for change once visualised artistically [43]. Given growing expectations that researchers account for the real-world impact of their research, introducing early career researchers to creative involvement strategies can bolster their preparedness for work in the research ecosystem whilst simultaneously improving that ecosystem’s contributions to the community.

Building on a foundation of interdisciplinary collaborations, these projects blend artistic creation and exploration with scientific endeavours to enhance science communication and impact within the general public [44]. Such collaboration is particularly crucial in our post-digital society; while existentialism thrives and human connection becomes increasingly entangled with (or disturbed by) technology, re-establishing humanity in research is of vital importance. While this challenge confronts all researchers, it can be particularly the case for early career researchers who may lack extensive experience or established PPI networks to draw on for grounding and guidance. Coordination of funding opportunities e.g. Research Ireland [45], the Basic Income for the Arts scheme [46] and the Science, Technology & Arts Initiative [47], enables cross-disciplinary collaboration between the sciences and the arts to facilitate valuable, sustainable and creative PPI partnerships from the early career stage onward.

For early-career researchers, creative involvement strategies provide a scaffolded learning opportunity where the inherent messiness of these approaches to involvement fosters adaptability and methodological breadth. As Phillips et al. (2024) note, navigating the broad spectrum of creative involvement strategies builds vital professional judgment [37]. Aligning with the Vitae Researcher Development Framework (2025), creative engagement cultivates personal qualities, such as curiosity, adaptability, and reflexivity, alongside professional competencies like confidence in engagement and public influence [48]. Demystifying creative collaboration with PPI contributors early on can help early-career researchers overcome the imposterism often felt during initial PPI forays, replacing apprehension with a robust, creative skillset.

## Navigating PPI data governance as an early-career researcher

A critical, yet often overlooked, issue in PPI research involves two distinct governance challenges: data sovereignty for PPI contributors and the ethical stewardship of research participants data. First, regarding the contributor data sovereignty, a challenge is the lack of data ownership among contributors. Although PPI contributors do not create participant data, they do generate key intellectual contributions through their decision-making roles; shaping research questions, methodological decisions, and sharing lived experience insights. This raises questions about ownership, as PPI contributors are often key to both developing and interpreting research results. Second, regarding participant data stewardship, PPI panels are well positioned to provide ethical oversight, yet clear mechanisms for this governance are rarely established. While tools to help guide PPI research exist, such as Richards et al. (2020) guidelines on academic authorship [49] and acknowledgment of patient partners in patient-oriented research, and the Contributor Role Taxonomy (CRediT) tool [50], a framework outlining the governance mechanisms over data ownership and stewardship is needed. These dual challenges are further compounded by the perceived complexity of data governance. Data Governance, as defined by the Data Management Body of Knowledge (DAMA), is the exercise of authority and control of data assets [51]. While the concept derives from IT systems management, certain principles can be easily applied to PPI research to establish a framework that clarifies both ownership for PPI contributors and stewardship for participant data, to facilitate more equitable collaboration.

**Fig. 3:**
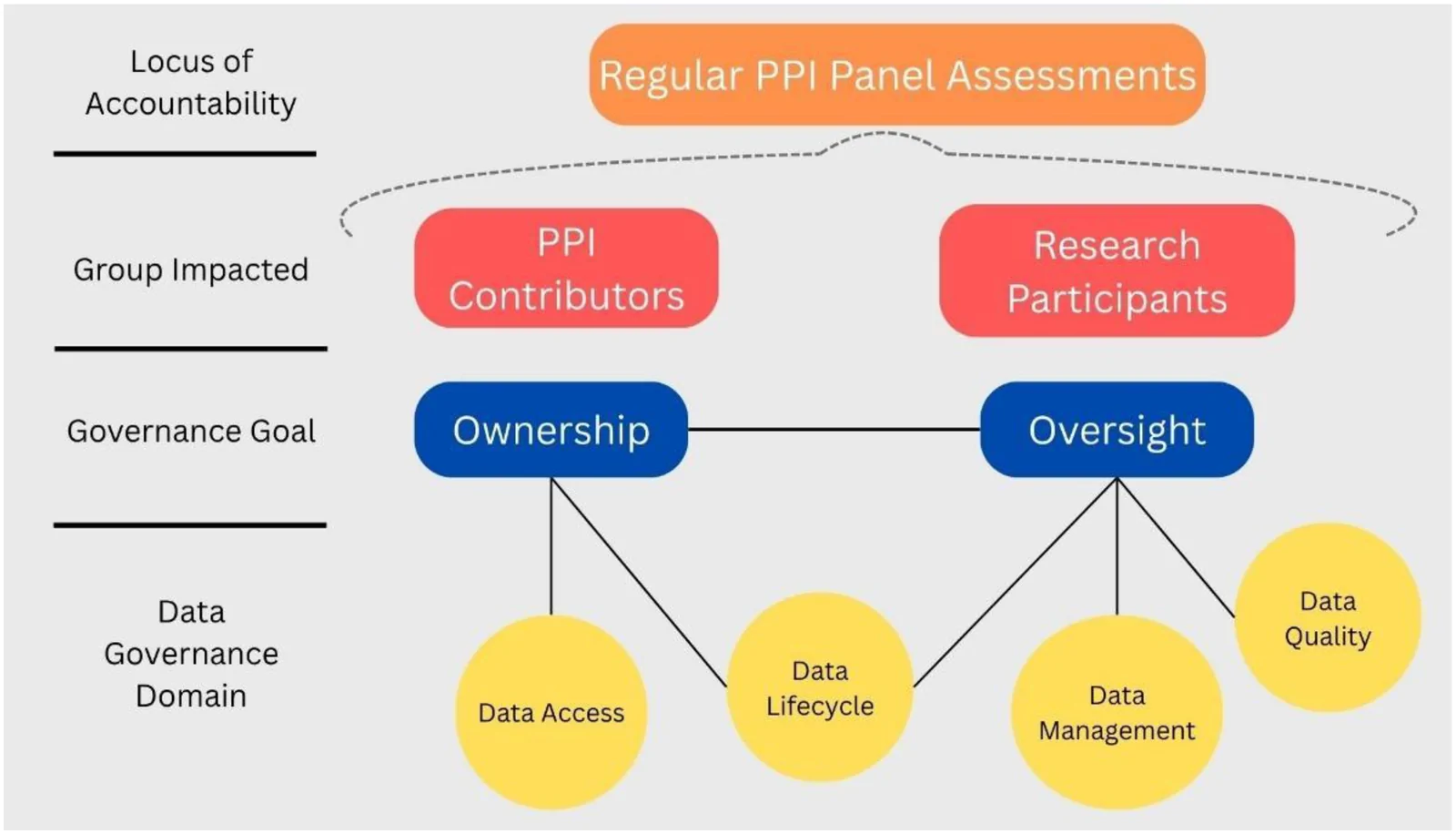
Data Governance Framework for PPI in Research

However, implementing effective data governance can be a daunting prospect due to the perceived complexity and time-consuming nature of data governance practices [52], especially for early-career researchers. Without a flexible adaptable framework, completing ethical PPI research can be challenging. Through identifying a framework for determining the ownership of contributions throughout PPI collaboration and forming a PPI panel to provide stewardship for research participant data, researchers can ensure their work is both ethical and transparent [53]. A novel framework consisting of data governance principles can serve to advance the knowledge and technical skills of early-career researchers, allowing PPI contributors to retain data sovereignty over their contributions and assist in the ethical management of research participant data. On this basis, we propose a governance framework that provides early-career researchers with a flexible template for ensuring PPI contributors’ data sovereignty and the ethical stewardship of research participant data. Drawing on existing data governance domains, this new framework outlines two overarching data governance goals within PPI: Ownership and Oversight, while outlining the overarching locus of accountability over decision-making, derived from Khatri and Brown (2010) alongside PPI panel assessments [54]. Placing PPI contributors at the core of the data management plan ensures both data sovereignty and ethical stewardship for PPI contributors and research participants (Fig. 3).

The first goal of the PPI panel is to determine ownership for PPI contributors. The governance domains of Data Access and Data Lifecycle [54] provide a roadmap for achieving this goal. Through this structure, a PPI panel can define the access requirements to PPI-generated data, determining who can access and use the insights and outputs produced by contributors, while managing the Data Lifecycle through clear governance plans. For example, this includes determining how contributor outputs are inventoried throughout the research program for definition, production, retention, and retirement of data [54]. This approach ensures transparency and grants PPI contributors true sovereignty over the material and intellectual products of their work.

The second goal of the PPI panel is to provide oversight for the ethical management of research participants’ data. PPI panels can conduct assessments to determine and update the Data Lifecycle, governing how participant information is produced, retained, managed and disposed of to ensure PPI contributors have full transparency and stewardship over the governance process [54]. Furthermore, PPI panels can guide safe management of research participant data by overseeing Data Management principles through the development of a program that protects participant data and information throughout its lifecycle [51]. This will ensure that research protocols are aligned to protect participant privacy throughout the project lifecycle [51], while acknowledging the necessity of consulting the relevant groups to identify additional requirements or expectations to achieve ethical and respectful participant data management across diverse groups. To this end, by defining Data Quality standards, PPI panels also ensure that research meets rigorous ethical benchmarks [51]. Regular assessment meetings ensure PPI contributors are integral to the governance process rather than being consulted sporadically; this prevents tokenism by embedding their expertise into the research infrastructure [53]. For early-career researchers, embedding PPI in research while adhering to rigorous ethical standards and accurately accrediting PPI ideation contributions during iterative and involved dialogical processes can be daunting. However, alongside guidelines for authorship and acknowledgement [49] and tools such as the CRediT taxonomy [50], our data governance framework offers a clear template for PPI contributors’ data sovereignty and ethical stewardship, providing much-needed guidance and clarity. Ultimately, this gives the next generation of researchers a robust platform from which to conduct meaningful, insightful, and ethically grounded research.

## PPI contributor perspective

Ethical and meaningful collaboration with PPI contributors cuts across the three key areas we have outlined; Pedagogy, Practice and Data Governance. Although PPI is complex and multi-faceted, as a PPI contributor, these are dimensions that are fundamental to facilitating a mutually respectful and beneficial collaboration. Specifically, although these points are relevant across the career-trajectories of researchers, I find them most relevant to early-career individuals not only because of unique pressures they face in beginning their careers but also because of their capacity to make mistakes in these areas and, importantly, to learn from them. As such, below I describe some of my own experiences as a PPI contributor across these areas while outlining suggestions about how some of these experiences could be improved by researchers going forward.

### Pedagogy

The ways that researchers learn about and do PPI directly influences how PPI contributors experience a project. I have collaborated with well-meaning researchers who have not been taught PPI as a core skill or have not been challenged to engage with this topic critically. This has led to an overly prescriptive approach to PPI being taken, where I as a contributor have felt pushed into pre-determined boxes and under-informed about my role. For early-career researchers in particular, this shows why PPI education needs to move beyond theory or compliance and prepare researchers to be adaptable and work collaboratively with contributors.

It can be beneficial for researchers to learn about PPI from the point of view of experienced contributors. I have facilitated upskilling workshops for researchers, developing my own skills while encouraging session participants to understand PPI from a new lens. For example, when I delivered a session focused on children’s participation in early years, I was able to share my own areas of knowledge and engage in critical dialogue and recontextualising some of my own ideas. Before this session, I was relatively unfamiliar with early years, but hearing directly from researchers helped me to rethink some of my assumptions and priorities. We shared reflections, examples, and where appropriate, disagreed constructively.

Collaboration within the learning stage, including through the development and use of PPI contributor-authored resources, can humanise PPI by showing what works well, what needs to improve, and what contributors may need in order to engage confidently. However, this should work both ways. PPI contributors should also be supported to understand what research is, what PPI can look like, and how their involvement may shape a project. This helps ensure that contributors are informed when agreeing to take part and are able to participate in a way that works for them.

This kind of exchange matters. When researchers and PPI contributors can learn from one another, it becomes easier to build an authentic culture of respect, curiosity and meaningful involvement.

### Practice

Reflecting on the projects that I have been involved in, it can be hard to differentiate one from the other as most follow a very similar structure centred on traditional consultative involvement. Consultation fatigue is real. Sitting in focus groups or online calls being asked the same questions, in the same ways can get monotonous. It can also create a bias, favouring the input of those who are most comfortable sharing through verbal or written formats. The projects that stand out to me most are those that adopted creative involvement strategies and encouraged us to discuss more openly and move away from pre-conceived notions of what the research should look like.

A particularly useful activity involved designing and pitching how we would lead a research project. It enabled us to think openly and share in our preferred formats, without the confines of specific questions or expected session outcomes. We then collaborated more consciously with the researchers to start thinking about our priorities, what was possible, and how we think the processes we had described should look. We designed slides, posters, and infographics that showed why we were championing certain ideas, but instead of feeling like we had to convince the researchers that our perspectives had merit, we were able to translate these thoughts into something real.

This shows the value of creative methods in PPI. They can make contributors’ thinking visible, support shared decision-making, and help researchers collaborate meaningfully and openly with contributors in ways that standard consultation strategies may not allow.

### Data governance

Data governance can matter deeply to PPI contributors. For some contributors, how their ideas are used and recognised is closely connected to whether they feel their contribution has been valued. There is no one-size-fits-all approach. What contributors need in order to feel appreciated within a team can vary between projects and may depend on time commitments, the focus of the work, the level of responsibility involved, and personal preferences. This is why conversations about authorship, ownership and recognition need to happen early and continue as projects develop.

One project in particular taught me this clearly. I was initially approached to take on a leadership role, including project management and primary writing responsibilities for a publication. The request was informal, with no contract, role description, or early dialogue about authorship, ownership or compensation. As a young person, it was exciting to be trusted within a professional environment, but these spaces were new to me. I did not know what questions I should ask, what was reasonable to expect, or what protections should have been in place. Data governance is not only a technical issue; it is also part of how trust, transparency and fairness are built within PPI relationships.

As the project developed, the lack of clear foundations became more difficult. Authorship and ownership were not discussed until late in the process, despite the level of responsibility involved. This recognition mattered to me because it was tied to how I understood the value of my contribution. I was eventually able to negotiate primary authorship, ensuring my contributions carried the same publication rights as those of academic colleagues.

The lesson is not that every contributor will want the same form of recognition. Rather, these conversations should be valued, prioritised and revisited as roles change. I have had more positive experiences where authorship and ownership were discussed early, recognition was proportional to contributions, and concerns were addressed quickly and respectfully. Good data governance does not need to be intimidating, but it does need to be intentional. Contributors should understand how their ideas may be used, how their role will be recognised, and what will happen if their responsibilities change. For early-career researchers, building these conversations into PPI from the beginning, and ensuring they continue throughout a project, can help prevent tokenism and support more ethical, transparent and mutually beneficial collaboration.

### Final thoughts

Meaningful PPI means moving from doing research on the public to doing research with the public, recognising the value contributors bring at every stage. This should shape how PPI is taught, practised and managed. Instilling this in researchers from an early stage in their career is an investment into a brighter future of meaningful collaboration between them the public, and patients. PPI is an ongoing journey, and for me, it works best when researchers and contributors navigate that journey together.

## Conclusion

From our perspectives as early-career researchers and a PPI contributor, pedagogy, practice and data governance form a key tripartite foundation for navigating the challenges of conducting meaningful PPI at an early stage in one’s career. We suggest several ways in which these challenges may be addressed by early-career researchers and those who guide them, with examples of effective and ineffective PPI provided by a PPI contributor themselves. We hope that these possible solutions can serve as a starting point to encourage further research in this area and greater uptake of PPI, vital steps for the next generation of researchers who will create the future of PPI.

## Data Availability

No datasets were generated or analysed during the current study.

## Abbreviations

PPI: Public and Patient Involvement
CIHR: The Canadian Institutes of Health Research
PCORI: The US Patient-Centred Outcomes Research Institute
IPPIN: the International Patient and Public Involvement Network
FOAMed: Free Open Access Medical Education
DAMA: Data Management Body of Knowledge
